# Data-driven coarse graining of large biomolecular structures

**DOI:** 10.1371/journal.pone.0183057

**Published:** 2017-08-17

**Authors:** Yi-Ling Chen, Michael Habeck

**Affiliations:** 1 Statistical Inverse Problems in Biophysics, Max Planck Institute for Biophysical Chemistry, Am Fassberg 11, 37077 Göttingen, Germany; 2 Department of NMR based Structural Biology, Max Planck Institute for Biophysical Chemistry, Am Fassberg 11, 37077 Göttingen, Germany; 3 Felix Bernstein Institute for Mathematical Statistics in the Biosciences, Georg August University Göttingen, Goldschmidtstrasse 7, 37077 Göttingen, Germany; Virginia Commonwealth University, UNITED STATES

## Abstract

Advances in experimental and computational techniques allow us to study the structure and dynamics of large biomolecular assemblies at increasingly higher resolution. However, with increasing structural detail it can be challenging to unravel the mechanism underlying the function of molecular machines. One reason is that atomistic simulations become computationally prohibitive. Moreover it is difficult to rationalize the functional mechanism of systems composed of tens of thousands to millions of atoms by following each atom’s movements. Coarse graining (CG) allows us to understand biological structures from a hierarchical perspective and to gradually zoom into the adequate level of structural detail. This article introduces a Bayesian approach for coarse graining biomolecular structures. We develop a probabilistic model that aims to represent the shape of an experimental structure as a cloud of bead particles. The particles interact via a pairwise potential whose parameters are estimated along with the bead positions and the CG mapping between atoms and beads. Our model can also be applied to density maps obtained by cryo-electron microscopy. We illustrate our approach on various test systems.

## Introduction

Biomolecular processes occur on many spatial and temporal scales [1]. An expanding array of experimental methods allows us to study the structure and dynamics of biological systems with increasing throughput and precision. Nevertheless computer simulations must often complement experiments to gain a quantitative understanding of the biological mechanism.

Molecular dynamics (MD) has developed into a powerful tool to study biomolecular systems with atomic detail [2, 3]. But typically there is a gap of several orders of magnitude between the atomic scale and the length and time scales that are biologically relevant. Therefore the computational burden posed by atomistic simulations becomes prohibitive for large biomolecular systems such as protein complexes. A remedy is provided by coarse graining (CG) approaches that reduce the system’s complexity by lumping together atoms into pseudo-atoms or beads [4–6].

Principled approaches to coarse graining start from an atomic potential such as an MD force field and try to derive transferable potentials that reproduce the thermodynamic and kinetic properties of the system as accurately as possible. CG methods that follow this approach include force matching [7], reverse and inverse Monte Carlo [8–11], and other methods for finding transferable CG potentials [12].

More pragmatic approaches derive a CG model directly from an experimental structure. Among the most popular methods are elastic network models [13, 14] that reduce the full structure to C*α* atoms and introduce harmonic springs between pairs of CG sites that are in contact. Network models were shown to reproduce the large-scale conformational dynamics of biomolecules. Recently, Xia and coworkers have developed a more principled approach that optimizes a CG model so as to reproduce the dynamic properties of a high-resolution elastic net [15, 16].

CG models are also used to interpret structural data. Due to a lack of resolution bead models constitute the most detailed 3D information that can be derived from small-angle X-ray scattering curves [17, 18], in case no additional high-resolution information is available. Another important application area is cryo-electron microscopy (cryo-EM). Three-dimensional reconstructions obtained from single-particle studies of biomolecular assemblies can be represented as bead models [19, 20]. These models have been used, for example, to predict the dynamics of biomolecular assemblies [21] or for rigid docking [22]. Recently, bead models were also used to obtain initial 3D reconstructions in single-particle analysis of projection images from cryo-EM [23].

There are two fundamental challenges in deriving CG models of biomolecular systems [5]. The first challenge is that the optimal choice of the CG sites, the so-called *CG mapping*, is in general unknown. Many CG methods for biomolecules such as proteins define beads along the amino acid sequence. For example, the MARTINI force field represents every amino acid by two beads, one for the backbone, one for the side chain [24]. However, if we want to represent many atoms by a single spherical bead, it will no longer be adequate to define CG groups along the polypeptide chain. Rather we need to combine atoms that are approximately enclosed in a sphere into a single CG particle, independent whether the atoms are part of the same or different amino acids. So the question of what is the best mapping between atoms and CG particles becomes highly relevant in cases where one does not want to use sequence information such as in *ultra-CG* pioneered by Voth and coworkers [25, 26]. Another reason might be that we are only given low-resolution data, which do not allow us to derive sequence information.

The second challenge is that the effective potential between CG particles is generally unknown. In network models, the potential form is simply imposed and mostly justified by pragmatic success [5]. However, a couple of more principled approaches such as the Yvon-Born-Green method [7, 12] allow us to estimate CG potentials that reproduce the properties of the thermodynamic ensemble. When constructing CG models from cryo-EM maps, pairwise interactions between the beads are typically ignored altogether [19, 20, 23].

Here we use Bayesian inference to develop a CG approach that is both principled and pragmatic. Our CG model does not incorporate any sequence information and represents the structure as a cloud of beads of equal size and occupancy. We introduce a Markov chain Monte Carlo (MCMC) algorithm for inferring the model from experimental structures. In addition to the CG mapping, our algorithm also learns the positions of the CG particles and the parameters of an interaction potential that regularizes the local structure of the bead model. Our model ignores any sequence information and can therefore be applied to atomic resolution structures as well as volumetric reconstructions from cryo-EM. The main application area will be ultra CG of large biomolecular assemblies rather than a detailed description of the molecular interactions. Therefore, we use only simple functional forms for the CG potential. We demonstrate our method on various protein complexes.

## Methods

Our goal is to learn a CG representation of a biomolecular system, including the force field and a CG mapping, from an experimental structure. Let us first assume that we are given an atomic-resolution structure (for example from the PDB [27]). Our probabilistic model aims to provide a quantitative answer to the following question: What is the best reduction of the structure to a much smaller collection of *K* spherical particles?

Our input data is an array of *N* three-dimensional atom positions ***x***_*n*_. We will denote the unknown positions of the beads by ***X***_*k*_ and demand that the CG structure not only approximates the atomic structure, but that it is also compatible with a CG force field *E*(***X***; **λ**) whose parameters we will estimate along with ***X***. We will use a Bayesian approach to infer the bead positions and all other unknown parameters.

### Mixture model approach to coarse graining

First, we need to formulate a probabilistic model that establishes a connection between the atomic structure (our data) and the CG structure (our unknown parameters). A simple approach is to view the input structure as a “cloud” of (fine-scale) atoms whose distribution in 3D space we describe with a Gaussian mixture model (GMM) [28]. According to the GMM, the probability of observing an atom at position ***x***_*n*_ is

$$\begin{array}{c} \mathrm{Pr}\left(x_{n}\;|\;X,s\right)=\frac{1}{K}\sum_{k=1}^{K}N\left(x_{n};X_{k},s^{2}\right) \end{array}$$ (1)

where $N(x_{n};\mu ,s^{2})$ is a 3D spherical Gaussian distribution

$$\begin{array}{c} N\left(x_{n};\mu ,s^{2}\right)=\frac{1}{\sqrt[{3}]{2\pi s^{2}}}\;\exp\{-\frac{1}{2s^{2}}{∥x_{n}-\mu ∥}^{2}\} \end{array}$$ (2)

scattering about a central location at ***μ*** with standard deviation *s*. The probability of the entire atomic structure ***x*** = (***x***_1_, …, ***x***_*N*_)′ is

$$\begin{array}{c} \mathrm{Pr}\left(x\;|\;X,s\right)=\prod_{n=1}^{N}\frac{1}{K}\sum_{k=1}^{K}N\left(x_{n};X_{k},s^{2}\right)\;. \end{array}$$ (3)

Given an atomic structure ***x***, we can find the optimal CG representation by maximizing the probability Eq (3) as a function of ***X*** and *s* (this function is also called the *likelihood function*). The likelihood function involves a product over sums, which cannot be optimized analytically. To find the CG model, we will use a trick that is typically applied in the estimation of GMMs.

We introduce binary *assignment* variables *Z*_*nk*_ ∈ {0, 1} that satisfy the constraints ∑_*k*_ *Z*_*nk*_ = 1. The assignment variables have the following meaning: If *Z*_*nk*_ = 1, the *n*-th atom at position ***x***_*n*_ will be replaced by the *k*-th bead at position ***X***_*k*_ in the CG representation. The constraints ∑_*k*_ *Z*_*nk*_ = 1 guarantee that every atom can only be assigned to exactly one bead. Therefore, the matrix ***Z*** encodes a mapping between atoms and beads.

With the help of the assignment variables, we can interpret Eq (1) as a marginal distribution:

$$\begin{array}{c} \mathrm{Pr}\left(x\;|\;X,s\right)=\sum_{Z}\mathrm{Pr}\left(x\;|\;X,Z,s\right)\times \mathrm{Pr}\left(Z\right) \end{array}$$ (4)

with the augmented likelihood Pr(***x*** | ***X***, ***Z***, *s*) (see S1 Appendix for details):

$$\begin{array}{c} \mathrm{Pr}\left(x\;|\;X,Z,s\right)=\frac{1}{\sqrt[{3N}]{2\pi s^{2}}}\;\exp\{-\frac{1}{2s^{2}}\sum_{k}N_{k}[∥X_{k}-\mu_{k}{∥}^{2}+s_{k}^{2}]\} \end{array}$$ (5)

and the summary statistics

$$\begin{array}{c} N_{k}=\sum_{n}Z_{nk},\;\mu_{k}=\frac{1}{N_{k}}\sum_{n}Z_{nk}x_{n},\;s_{k}^{2}=\frac{1}{N_{k}}\sum_{n}Z_{nk}{∥x_{n}-\mu_{k}∥}^{2}\;. \end{array}$$ (6)

*N*_*k*_ is the number of atoms that have been assigned to the *k*-th bead, ***μ***_*k*_ is the center of mass of the assigned atoms and *s*_*k*_ is the radius of the *k*-th cluster.

### Prior distribution over coarse-grained structures

Without any detailed prior about the CG positions, random sampling from the full posterior Pr(***X***, ***Z***, *s*|***x***) would be simply an exercise in density estimation with a GMM. However, there is no guarantee that bead configurations ***X*** will be physically realistic. We will therefore use a Boltzmann ensemble based on a force field *E*(***X***; **λ**) as a prior over the CG positions:

$$\begin{array}{c} \mathrm{Pr}\left(X\;|\;\lambda \right)=\frac{1}{Z(\lambda )}\exp\left\{-E\left(X;\lambda \right)\right\}\;. \end{array}$$ (7)

The parameters **λ** of the CG potential *E*(***X***; **λ**) are unknown and will be estimated along with the CG configuration. A major difficulty stems from the fact that we cannot evaluate the partition function

$$\begin{array}{c} Z(\lambda )=\int \exp\{-E(X;\lambda )\}\;dX \end{array}$$ (8)

analytically. We will therefore use the configurational temperature formalism [29] to estimate **λ** for given ***X***.

We expand the CG potential energy into a linear combination of *L* structural features *f*_*l*_(***X***) [29, 30]:

$$\begin{array}{c} E\left(X;\lambda \right)=\sum_{l=1}^{L}\lambda_{l}f_{l}\left(X\right)=\left\langle \lambda ,f(X)\right\rangle \;. \end{array}$$ (9)

Many molecular interaction potentials can be parameterized in this way. Typically, we will use only a small number of features as it is the case, for example, for the Lennard-Jones (LJ) potential:

$$\begin{array}{c} f_{l}\left(X\right)=\sum_{k<k^{\prime }}{∥X_{k}-X_{k^{\prime }}∥}^{-6l},\;l=1,2 \end{array}$$ (10)

with *L* = 2 features corresponding to the attractive and the repulsive part of the LJ potential.

### Inference of model parameters

We use Markov chain Monte Carlo (MCMC) [31] to infer the model parameters ***X***, ***Z***, *s*, and **λ**. Our MCMC strategy is a Gibbs sampler [32], which updates groups of parameters successively by drawing from the conditional posteriors:

$$X\;∼e^{-\frac{1}{2s^{2}}\Sigma_{k}N_{k}∥X_{k}-\mu_{k}{∥}^{2}-\langle \lambda ,f\left(X\right)\rangle }$$ (11)

$$Z∼M\left(1,p_{n}\right)$$ (12)

$$s^{-2}∼G\left(3N/2,\chi^{2}\left(X\right)/2\right)$$ (13)

$$\lambda ∼\frac{1}{Z\left(\lambda \right)}e^{-\langle \lambda ,f\left(X\right)\rangle }$$ (14)

where we introduced the Gamma distribution G(α,β) with shape parameter *α* > 0 and scale *β* > 0, the multinomial distribution $M(1,p_{n})$, the assignment probabilities

$$\begin{array}{c} p_{nk}=\frac{\exp\{-\frac{1}{2s^{2}}∥x_{n}-X_{k}{∥}^{2}\}}{\sum_{k^{\prime }}\exp\{-\frac{1}{2s^{2}}∥x_{n}-X_{k^{\prime }}{∥}^{2}\}} \end{array}$$ (15)

and the goodness of fit between the atomic structure and the CG model

$$\begin{array}{c} \chi^{2}\left(X\right)=\sum_{n,k}Z_{nk}{∥x_{n}-X_{k}∥}^{2}\;. \end{array}$$ (16)

In words, the Gibbs sampler progresses by successively updating the bead positions (Eq 11), the CG mapping (Eq 12), the precision of the CG model (Eq 13) and the parameters of the CG potential (Eq 14). Steps Eqs (12) and (13) are straight forward: We simply run random number generators for the multinomial distribution and the Gamma distribution to update the CG mapping ***Z*** and the precision *s*^−2^. To draw posterior samples of ***X*** and **λ** is more challenging.

### Posterior sampling of bead positions

Let us write the conditional posterior of the CG positions (Eq 11) in canonical form:

$$\begin{array}{c} \mathrm{Pr}(X\;|\;Z,s,\lambda ,x)\propto \exp\{-U(X)\} \end{array}$$

where the “potential energy” function *U*(***X***) is the sum of a data-dependent term and the force field:

$$\begin{array}{c} U\left(X\right)=\frac{1}{2s^{2}}\sum_{k}N_{k}{∥X_{k}-\mu_{k}∥}^{2}+\left\langle \lambda ,f(X)\right\rangle \;. \end{array}$$ (17)

The data-dependent term pulls the CG beads towards the cluster centers ***μ***_*k*_ with a harmonic force, but the CG potential *E*(***X***; ***λ***) prevents clashes between beads and regularizes the local structure resulting in a local order that is similar to a fluid. We use Hamiltonian Monte Carlo (HMC) [33, 34] to generate configurations from exp{−*U*(***X***)} (see S1 Appendix for details).

### Estimation of the CG potential

The conditional posterior distribution of the force field parameters **λ** depends only on the CG structure (Eq 14). A major complication is posed by the intractability of the normalizing constant *Z*(**λ**). Because we cannot compute *Z*(**λ**) analytically, it is not possible to use a standard Monte Carlo algorithm to estimate **λ**, which would require knowledge of *Z*(**λ**) to evaluate the acceptance probability. Monte Carlo algorithms that deal with the intractability of the partition function have been proposed (see e.g. [30, 35]), but these algorithms either make assumptions that are not satisfied in our context or suffer from slow convergence. We therefore use a simple approximation based on the configurational temperature formalism [29].

In brief, we consider the CG parameters **λ** as a set of *L* inverse temperatures that can also adopt negative values. Given the current CG positions ***X***, we compute an *L* × *L* matrix ***A*** and an *L*-vector ***b*** as follows:

$$\begin{array}{c} A_{ll^{\prime }}\left(X\right)={\left[\nabla f_{l}\left(X\right)\right]}^{\prime }\left[\nabla f_{l^{\prime }}\left(X\right)\right],\;b_{l}\left(X\right)=\Delta f_{l}\left(X\right) \end{array}$$ (18)

where **∇***f*_*l*_ is the gradient of the *l*-th feature with respect to the bead positions and Δ = **∇**′**∇** is the Laplace operator. We then solve the system of linear equations

$$\begin{array}{c} A(X)\;\lambda =b(X) \end{array}$$ (19)

to determine our next estimate of **λ**. We can restrict the parameters of the CG potential by introducing a prior probability Pr(**λ**) such as, for example, a zero-centered Gaussian or a Laplacian prior favoring sparse parameterizations and update **λ** by minimizing [29]

$$\begin{array}{c} \frac{1}{2}{∥A\left(X\right)\;\lambda -b\left(X\right)∥}^{2}-\log\mathrm{Pr}\left(\lambda \right)\;. \end{array}$$ (20)

### Reordering beads to preserve local structure

The mixture model (Eq 3) as well as the prior (Eq 7) are invariant under permutation of the bead indices *k* = 1, …, *K*. We can therefore renumber a given configuration of the beads ***X*** such that spatially close beads have similar indices. Due to the permutation invariance renumbering the beads will not change the posterior probability of the configuration. To find a meaningful order of the beads that reflects the local structure, we find the shortest path that visits every bead exactly once, based on the Euclidean distance matrix ∥***X***_*k*_ − ***X***_*k*′_∥ as a cost function. By solving this traveling salesman problem, we find a permutation of the beads that preserves the local connectivity as much as possible.

### Python implementation

Our coarse-graining algorithm has been implemented as a Python/Cython library that depends only on freely available libraries including numpy, scipy and csb. The library can be cloned or downloaded at https://github.com/michaelhabeck/cg.

## Results and discussion

We ran our Bayesian CG algorithm on several large biomolecular assemblies some of which served as a benchmark previously [16].

### Coarse-grained model of Arp2/3

Let us first look at a particular assembly in more detail, the Arp2/3 complex. Arp2/3 is an asymmetric heteromer composed of seven chains and has a total molecular weight of 224 kDa. We ran our algorithm with *K* = 500 beads to find a CG representation of the crystal structure (PDB code 1tyq). The crystal structure comprises 13341 heavy atom positions, which we approximate by CG particles (without taking the mass differences between atoms of different types into account). Therefore, on average a bead will represent *N*/*K* ≈ 26 atoms.

**Fig 1:**
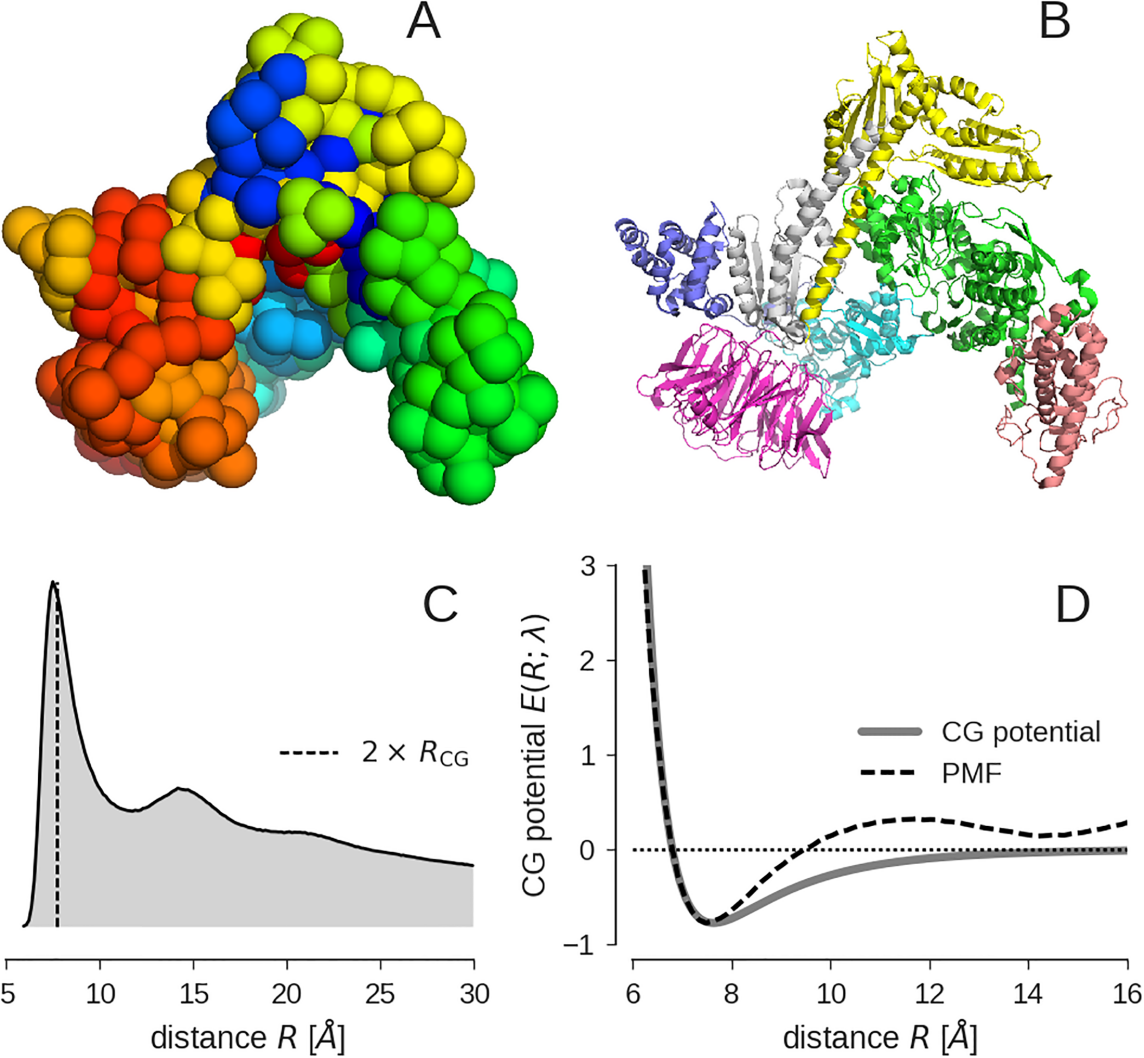
CG representation of the Arp2/3 complex. **(A)** Space filling representation of the 500-bead model with sphere radius *R*_CG_ ≈ 3.82 Å. **(B)** Cartoon representation of the crystal structure of Arp2/3 (PDB code 1tyq) with chains highlighted in different colors. The PDB entry provides structural information for a total of 1692 amino acids and 13341 heavy atoms. **(C)** Radial distribution function (RDF); the dashed vertical line indicates 2 × *R*_CG_. **(D)** Potential of mean force (PMF) obtained by Boltzmann inversion of the RDF (dashed line) compared to the estimated CG potential (gray solid line).

Fig 1A shows a representative structure that we generated from the posterior (Eq 11); it reproduces the shape of the crystal structure (Fig 1B). The size of the spheres indicates the inferred van der Waals radius of the beads *R*_CG_ ≈ 3.82 Å, defined by the minimum of the estimated LJ potential. The radial distribution function (RDF) reflects the local packing order of the bead model (Fig 1C). The RDF of the Arp2/3 model bears some similarity to an RDF of a monatomic fluid. The first peak indicates an accumulation of neighbors at 2 × *R*_CG_ due to first-shell interactions. This local order is rapidly lost, which is indicated by subordinate peaks in the RDF that are located at higher-order shells similar to a fluid. Upon Boltzmann inversion of the RDF, we obtain a potential of mean force (PMF), which we can compare to the estimated CG potential (Fig 1D). The PMF of a CG particle system is used in the Boltzmann inversion method [10] as an initial guess for the effective pair interaction potential between the beads. There is a clear difference between the PMF and the CG potential. Whereas the CG potential has only a single minimum at 2 × *R*_CG_, the PMF shows multiple minima.

### Impact of the CG prior

Let us next study the impact of the CG potential and the Boltzmann prior on the bead model. To do so, we also ran the Gibbs sampler on a posterior whose CG parameters **λ**_*l*_ were clamped to zero. The overall size of the CG structure is very similar with a radius of gyration of *R*_g_ = 43.19 ± 0.02 Å (with Boltzmann prior) and *R*_g_ = 42.70 ± 0.02 Å (without Boltzmann prior) and close to the size of the input structure *R*_g_ = 43.15 Å. Also the estimated precision of the models with and without the Boltzmann prior is very similar: *s* = 2.43 ± 0.01 Å (with Boltzmann prior) and *s* = 2.44 ± 0.01 Å (without Boltzmann prior). The introduction of the particle interactions does not compromise the accuracy of the CG model.

**Fig 2:**
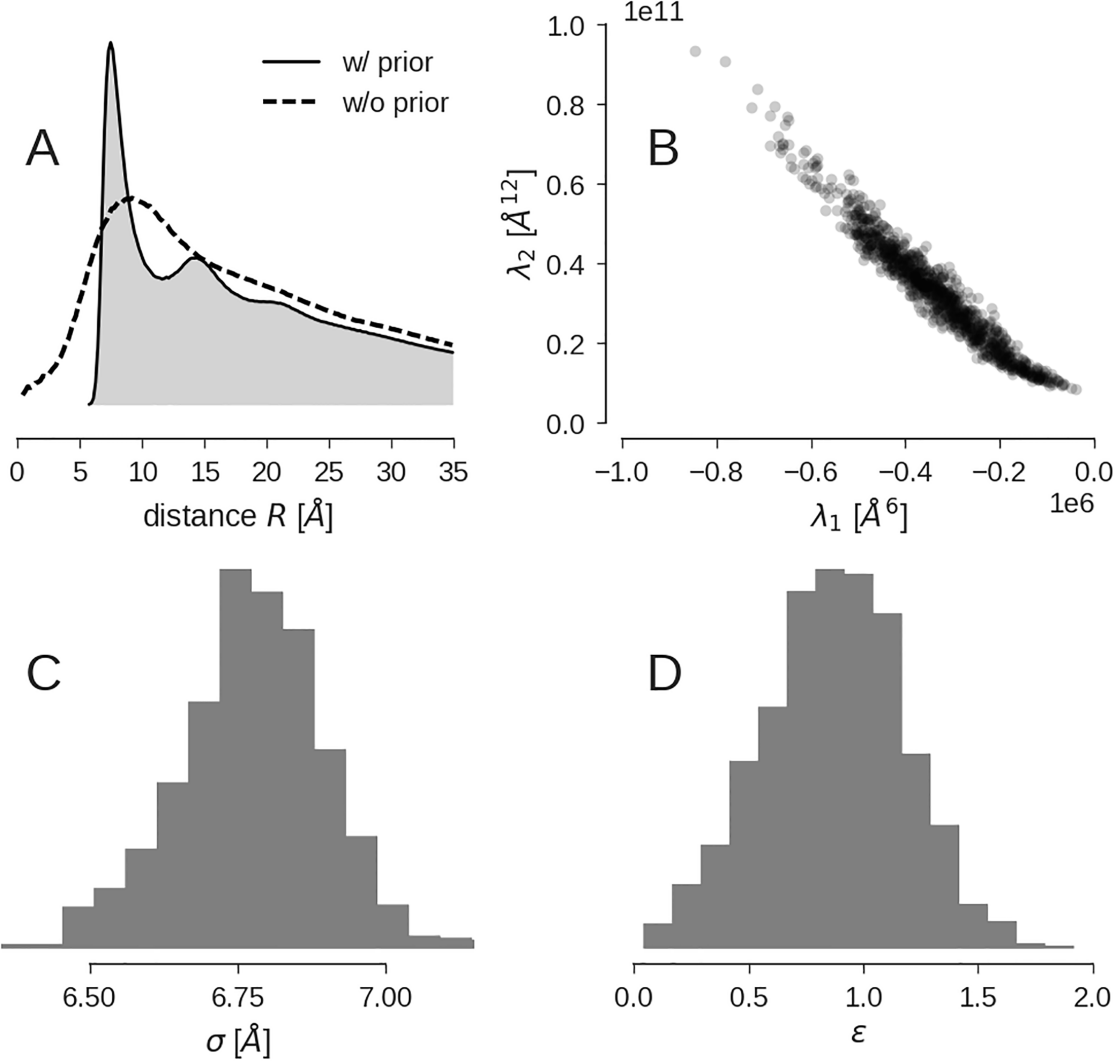
Impact of the CG potential *E*(*X*; λ) on the 500-bead model of Arp2/3. **(A)** RDF of the models computed with Boltzmann prior (solid line, grey fill) and without the Boltzmann prior (dashed line). **(B)** Sampled force field parameters λ_1_ and λ_2_. **(C)** Posterior distribution of the interaction range *σ*. **(D)** Posterior distribution of the depth of the potential well $\epsilon =\lambda_{1}^{2}/4\lambda_{2}$.

In Fig 2A we show the RDFs obtained with and without the Boltzmann prior (Eq 7). Without the introduction of the Boltzmann prior the configurations of the CG model are more flexible and produce configurations in which beads come significantly closer to each other than in the regularized CG models. The RDF exhibits a single peak which is much broader and shifted to a larger value compared to the RDF obtained in the presence of the Boltzmann prior. Only with the introduction of the Boltzmann prior, the CG model exhibits the desired local order.

Fig 2B shows the sampled parameters **λ**_1_ and **λ**_2_ of the CG potential. The parameters fluctuate significantly but scatter about stable averages. It is convenient to map the parameters to the standard parameterization of the Lennard-Jones potential $\sigma =\sqrt[{6}]{-\lambda_{2}/\lambda_{1}}$ and $\epsilon =\lambda_{1}^{2}/4\lambda_{2}$ where $R_{\text{CG}}=\sqrt[{6}]{2}\;\sigma /2$. The posterior distributions of these parameters are shown in Fig 2C and 2D, indicating that the Gibbs sampler produces well-defined approximations of the posterior distribution.

### Impact of the degree of coarse graining

**Fig 3:**
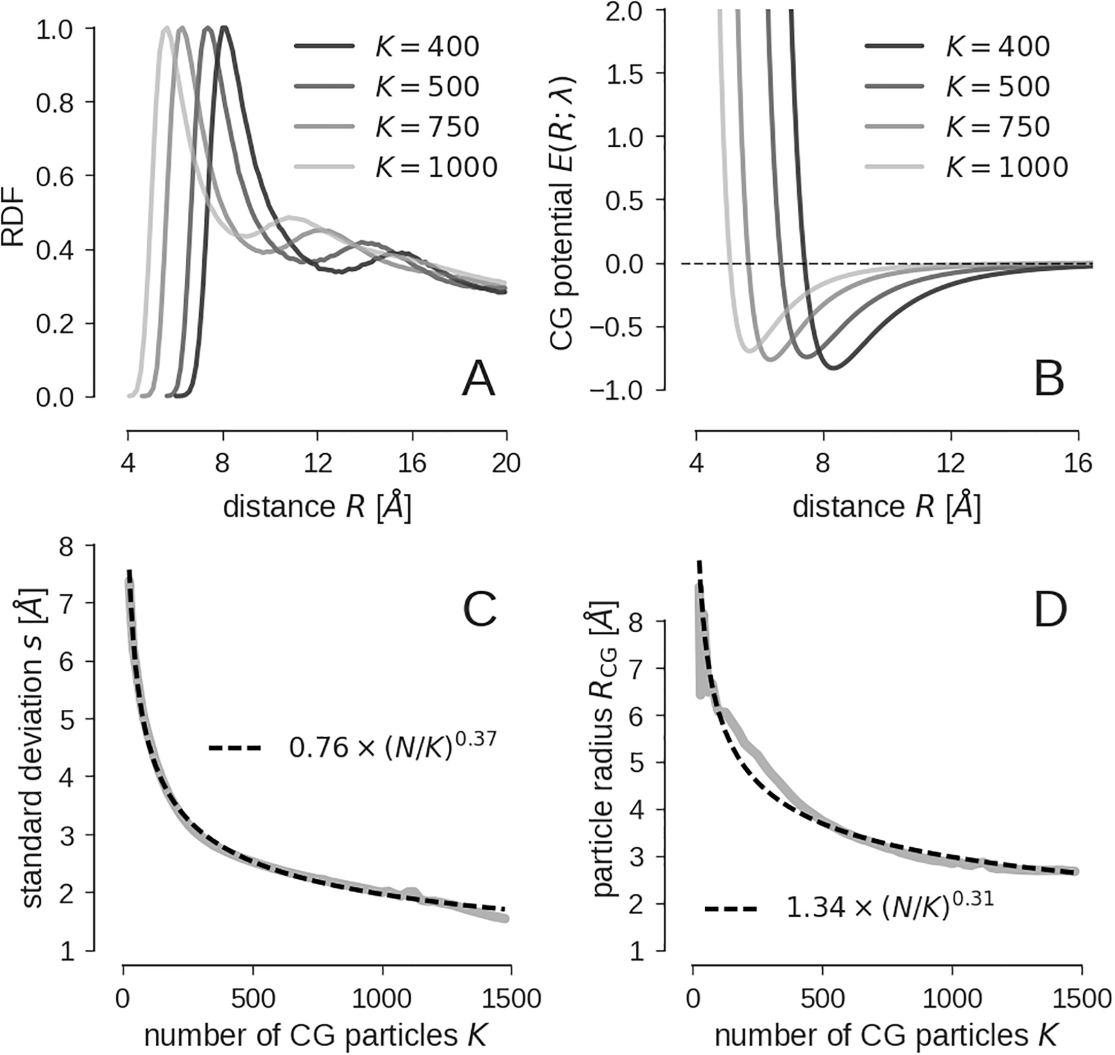
CG models at different levels of coarse graining. **(A)** RDF for different resolutions. **(B)** Estimated CG potentials. **(C)** Standard deviation (error of the CG model) *s* as a function of the number of coarse grained particles *K*. **(D)** Particle radius *R*_CG_ as a function of *K*. The dependence of *s* and *R*_CG_ on *K* is modeled with a power law. The empirical fits are shown as dashed black lines in panels **(C)** and **(D)**.

We ran the CG algorithm for different choices of *K* using the Arp2/3 complex as input structure. Representative bead models are shown in S1 Fig. We expect that with decreasing *K*, the radius of the CG particle increases, because more and more atoms (≈ *N*/*K*) are represented by a single CG particle. Fig 3A and 3B show the RDF for a different number of CG sites and the estimated Lennard-Jones potentials. As expected, the size of the CG particles decreases with increasing number of CG particles *K*, which is indicated by a shift of the first-shell peak in the RDF. A corresponding shift is observable in the minimum of the estimated CG potential. However, the strength of the CG potential is only weakly affected by changes in *K*.

We computed an entire series of CG models for Arp2/3 with *K* ranging from 25 to 1500. Fig 3C shows the error of the CG model *s*, which can also be seen as the resolution of the model. For smaller *s*, more and more details of the input structure are represented. By fitting a straight line between values of ln*K* and ln*s*, we obtain a power law for the dependency of the resolution and the number of bead particles (see dashed line in Fig 3C).

A similar behavior is found for the radius of the CG particle. Fig 3D shows that *R*_CG_ decreases as *K* increases. A hard sphere model would roughly give *R*_CG_ ≈ *r*(*N*/*K*)^1/3^ for the relation between the radius of the CG particle and the number of beads where *r* is the radius of the (fine-scale) atoms. We find that this relation is approximately fulfilled in our coarse grained models with an exponent of 0.31 instead of 1/3 and an atom radius of *r* = 1.34 Å, which is reasonable because the input structure is based on the positions of all heavy atoms, which have similar van der Waals radii.

To compare our CG models with alternative coarse-graining approaches, we also computed CG models of the Arp2/3 complex using the vector quantization approach implemented in *quanpdb* [36]. S2 Fig shows the RDFs obtained with our CG approach and quanpdb. Whereas the Bayesian models exhibit fluid-like packing marked by a first- and second-shell peak, the quanpdb models show a much broader RDF indicating a loose packing of the CG particles.

### Transferability of coarse graining parameters

**Fig 4:**
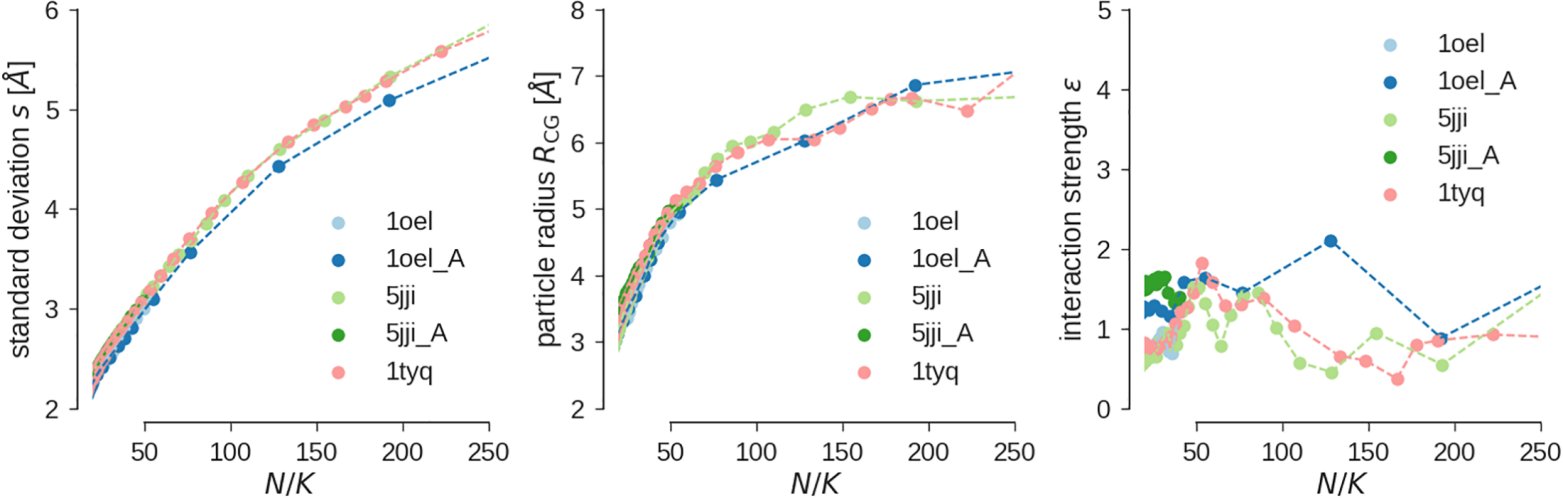
Transferability of CG potential. Error of the CG model **(A)**, CG particle radius **(B)** and interaction strength *ϵ* **(C)** for various biomolecular structures.

To study if the CG model is to some extent transferable, we also calculated various CG models for GroEL (PDB code 1oel, *N* = 26929) and Rho transcription termination factor (PDB code 5jji, *N* = 19305), which are both symmetric assemblies. We computed CG models for a single subunit and for the entire assemblies for a large number of CG particles. Fig 4 shows how the error of the model *s*, the radius of the CG particles *R*_CG_ and the depth of the potential well *ϵ* depend on the average number of atoms per bead *N*/*K* for the different structures. Overall, we see a large agreement in the behavior of these parameters, which argues for the transferability of our simple CG model. The error of the model and the bead radius show the same dependence on *N*/*K* as for the Arp2/3 complex (see Fig 3). A larger variability is observed for the interaction parameter *ϵ* (Fig 4C), which scatters between 0.5 an 2.0 for the different assemblies and number of CG particles. More examples of coarse-grained models of large assemblies can be found in S3 Fig.

### Coarse graining of EM maps

**Fig 5:**
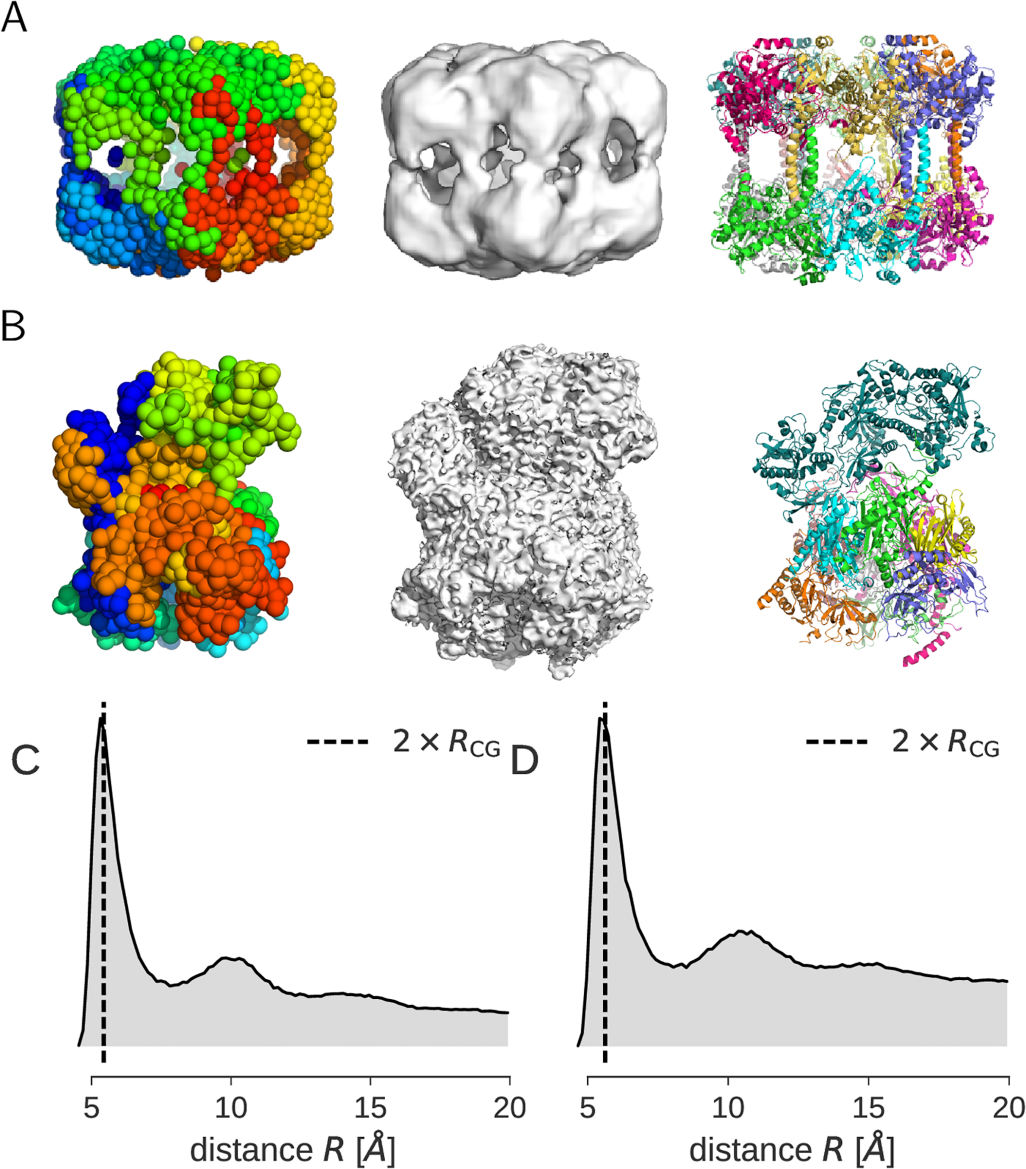
CG models of Lengsin and the exosome derived from cryo-EM maps. The EM map EMD-1290 shows Lengsin at 17 Å resolution and was used to derive a CG model comprised of 2000 beads. The EM map EMD-3366 shows the exosome at 4.2 Å resolution and was used to derive a CG model comprised of 2000 beads. **(A)** Side view of the CG model of Lengsin (left), EM map (middle) and the atomic structure (PDB code 2j9i) (right). **(B)** Side view of the CG model of the exosome (left), EM map (middle) and the atomic structure (PDB code 5g06) (right). The CG particles were sorted so as to minimize the path through all bead positions and are colored from blue to red. The radial distribution functions of the CG models are shown in panels **(C)** Lengsin and **(D)** exosome.

It is also possible to fit CG models to 3D reconstructions obtained with cryo-electron microscopy (cryo-EM). A detailed derivation can be found in the S1 Appendix. To illustrate coarse graining of cryo-EM maps, we used a reconstruction of Lengsin at 17 Å resolution (EMD-1290) [37] and a reconstruction of the exosome at 4.2 Å resolution (EMD-3366) [38]. We used *K* = 2000 particles to approximate both EM reconstructions. The CG models are shown in Fig 5 together with the EM map and an atomic structure of Lengsin and the exosome. The estimated particle radii are *R*_CG_ = 2.7 Å for the Lengsin model and *R*_CG_ = 2.8 Å for the exosome.

The Xmipp package for cryo-EM data processing [39] provides a method for computing particle based CG models from 3D maps [20]. We ran Xmipp’s CG procedure with default settings on the density map of the exosome (EMD-3366). Xmipp produced a CG model comprised of 2308 CG particles. The maximum cross-correlation coefficient (CC) between the EM map and the CG model is 55%, which is significantly smaller than CC obtained with our CG model: CC = 73%. The RDF of the Xmipp model shows a strong peak at very small distances indicating that the pseudoatoms are not packed in a physically realistic fashion (see S4 Fig).

### Modeling conformational dynamics

CG models are often used to predict conformational dynamics (e.g. [20, 40]). The most common approach is based on elastic network models [13, 14]. We tried to predict the structural dynamics of adenylate kinase (AK), which is a standard test system to study conformational changes in proteins. AK adopts an open conformation in the unbound state and a closed conformation upon binding of two substrate molecules. We computed normal modes by diagonalizing the Hessian matrix derived from various network models. A common choice to define an anisotropic network model is to use C*α* positions and a cutoff distance of 15 Å [14]. To define the network based on our CG models of AK, we used a cutoff distance of 7 × *R*_CG_; we have not tried to optimize our cutoff criterion, and other choices might result in better predictions. To assess how well the network captures the experimentally observed conformational change, we computed the overlap between individual normal modes and the displacement vectors computed from the superimposed open and closed states of AK (analogous to the analysis in [20, 40]). We used the *ProDy* python package [41] to carry out the anisotropic network analysis.

**Fig 6:**
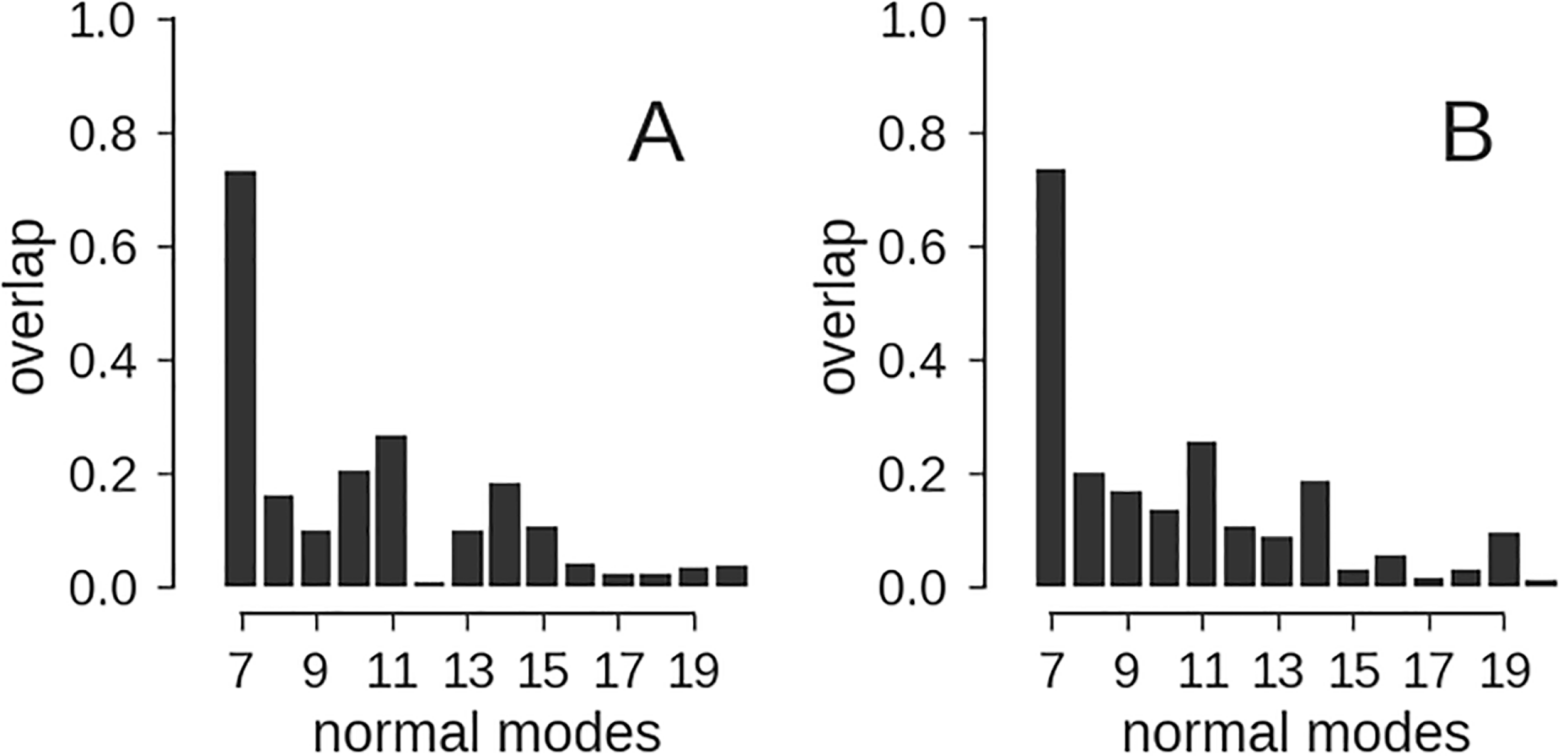
Normal mode analysis of adenylate kinase (AK). An anisotropic network model was derived from the C*α* positions of the open conformation of AK (PDB code 4ake) and used to compute normal modes. The overlap between the normal modes and the conformational change from the open to the closed structure of AK (PDB code 1ake) was computed as described by Stember and Wriggers [40] and is shown in panel **(A)**. The same analysis was carried out for a CG model comprised of *K* = 50 beads. To compute the overlap with the experimental conformational change, the normal modes derived from the CG model where interpolated using a thin-plate spline and are shwon in panel **(B)**.

Fig 6 shows the overlap of the first 14 normal modes and the open-to-closed transition. Both the C*α* model and the CG model with *K* = 50 beads show a similar pattern in the overlap values. The largest portion of the open-to-closed transition is sampled along the first non-trivial normal mode (mode 7). Indeed this mode represents the closure of the LID domain. The second most significant motion is captured by mode 11 according to both models. An animation of this mode reveals that it corresponds to movements of the NMP-binding domain. Thus the CG model is able to capture some aspects of AK’s conformational dynamics. With too few beads (*K* ≤ 30), we observed a breakdown of the predictive power of the CG model with regard to conformational dynamics, whereas the shape is still approximated well (see S5 Fig for details).

## Conclusion

This article introduces a data-driven Bayesian approach for coarse graining large biomolecular assemblies. In addition to the positions of the beads, our model estimates a mapping between atoms and beads as well as the parameters of a CG potential. The CG potential regularizes the local structure of the CG model, whereas the likelihood resulting from a Gaussian mixture model imposes overall shape restraints.

The main motivation for our coarse-graining algorithm is to model large biomolecular complexes in an integrative approach guided by cryo-EM maps, crosslinking data etc. In the simplest scenario, the subunits would be represented as coarse-grained structures that can only move rigidly. In combination with an elastic network approach, our CG models also seems to be suitable for predicting conformational dynamics as illustrated for adenylate kinase. This might be useful for flexible fitting of CG models.

With dreceasing number of beads, our models become very coarse: tens to hundreds of atoms are represented by a single bead. An obvious limitation of such ultra CG models is that it becomes impossible to model structural changes that occur within beads. Another drawback of our current model is that the CG potential is not physically realistic but mainly serves to improve the bead packing. Future extensions of our model could include more realistic force field terms that are able to capture some of the physico-chemical properties of biomolecular assemblies. Moreover, it might also be interesting to introduce multiple species of CG particles.

Our current model has a single unknown parameter, the number of beads *K*. In the future, we would like to develop methods that estimate the number of CG particles from structural data such as cryo-EM maps or solution scattering curves. Another future extension is to expand the current model into a hierarchy of CG models in which intermediate resolution structures serve as input structure for estimating a CG model with coarser resolution. The hierarchy of CG models could then be simulated by using resolution exchange Monte Carlo [42]. Other interesting extensions include the use of symmetry when coarse graining symmetric assemblies and integrative modeling of macromolecular complexes from cryo-EM and crosslinking / mass spectrometry.

## Supporting information

S1 Fig CG models of Arp2/3 with number of beads increasing from top to bottom and left to right. (PNG)

S2 Fig Comparison of our Bayesian coarse-graining method with *quanpdb* (vector quantization). CG models of Arp2/3 were computed for a varying number of beads (*K* = 400, 500, 750, 1000). Shown is the radial distribution function (RDF) obtained from the CG models. **(A)** Bayesian CG models. **(B)** quanpdb.
(PDF)

S3 Fig Coarse-grained models of large biomolecular assemblies. CG models of **(A)** F-actin (PDB code 3j8i, *N* = 14660, *K* = 250), **(B)** Rho transcription factor (PDB code 5jji, *N* = 19305, *K* = 500), **(C)** GroEL/ES (PDB code 1aon, *N* = 58674, *K* = 1000) and **(D)** of the 26S proteasome (PDB code 5t0c, *N* = 155216, *K* = 2000).
(PDF)

S4 Fig Comparison of our Bayesian coarse-graining method with Xmipp. Radial distribution function (RDF) of a CG model obtained from the exosome map using Xmipp’s volume-to-pseudoatom command. **(A)** The full RDF shows a dominant peak close to zero resulting from a few very small distances. **(B)** If we zoom into the RDF at larger distances, the RDF shows fluid-like features. However, the first- and second-shell peak are less pronounced than in the RDF resulting from our coarse-graining procedure.
(PDF)

S5 Fig Overlap between conformational change of adenylate kinase and normal modes calculated from various CG models. See the caption of Fig 6 for more details. The insets show that CG models that served as an input for the anisotropic network analysis. The lower right panel shows the overlap distribution of the C*α* model for comparison.
(PDF)

S1 Appendix Algorithmic details. (PDF)
