# Supplementary figures and images for: Data-driven coarse graining of large biomolecular structures
Source: PLoS One. 2017 Aug 17;12(8):e0183057. doi: 10.1371/journal.pone.0183057 (PMC5560709; doi:10.1371/journal.pone.0183057)

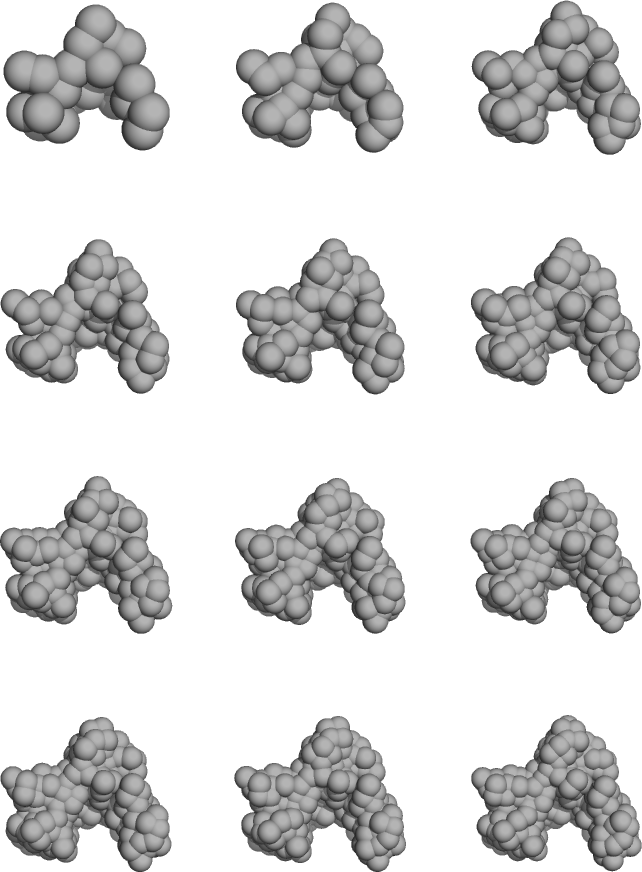

Supplement: S1 Fig — (PNG) [file pone.0183057.s001.png]

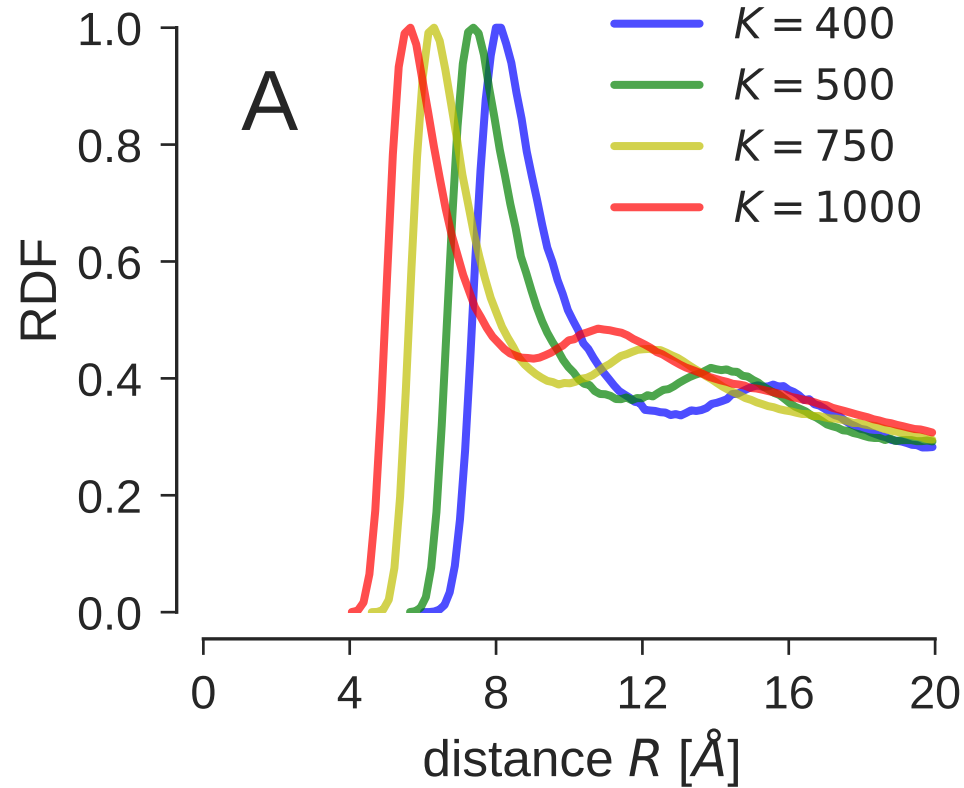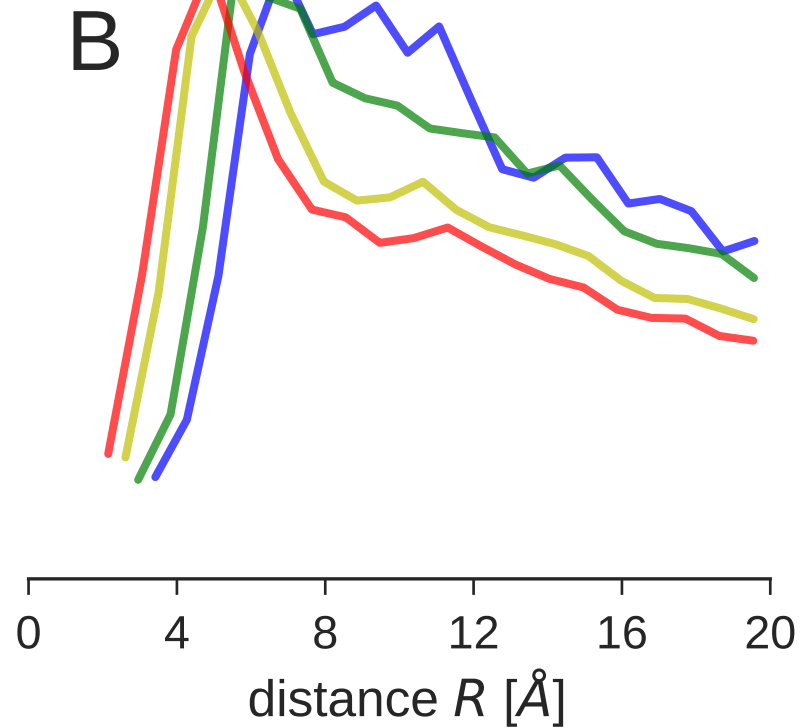

Supplement: S2 Fig — CG models of Arp2/3 were computed for a varying number of beads (K = 400, 500, 750, 1000). Shown is the radial distribution function (RDF) obtained from the CG models. (A) Bayesian CG models. (B) quanpdb. (PDF) [file pone.0183057.s002.pdf]

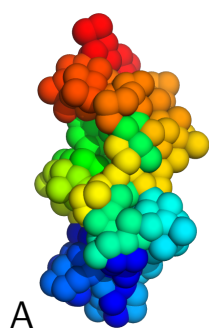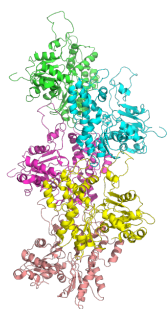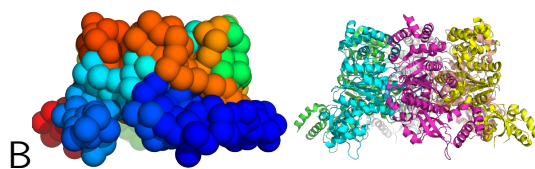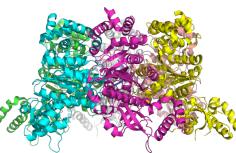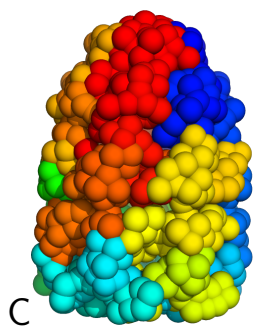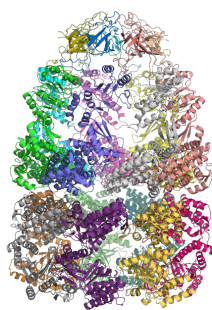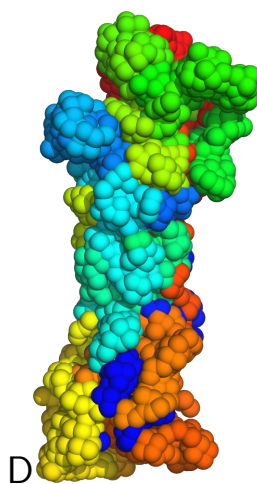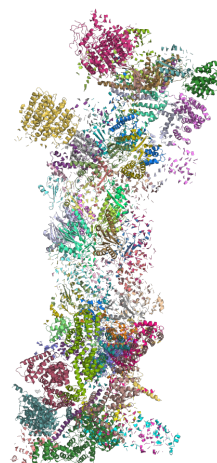

Supplement: S3 Fig — CG models of (A) F-actin (PDB code 3j8i, N = 14660, K = 250), (B) Rho transcription factor (PDB code 5jji, N = 19305, K = 500), (C) GroEL/ES (PDB code 1aon, N = 58674, K = 1000) and (D) of the 26S proteasome (PDB code 5t0c, N = 155216, K = 2000). (PDF) [file pone.0183057.s003.pdf]

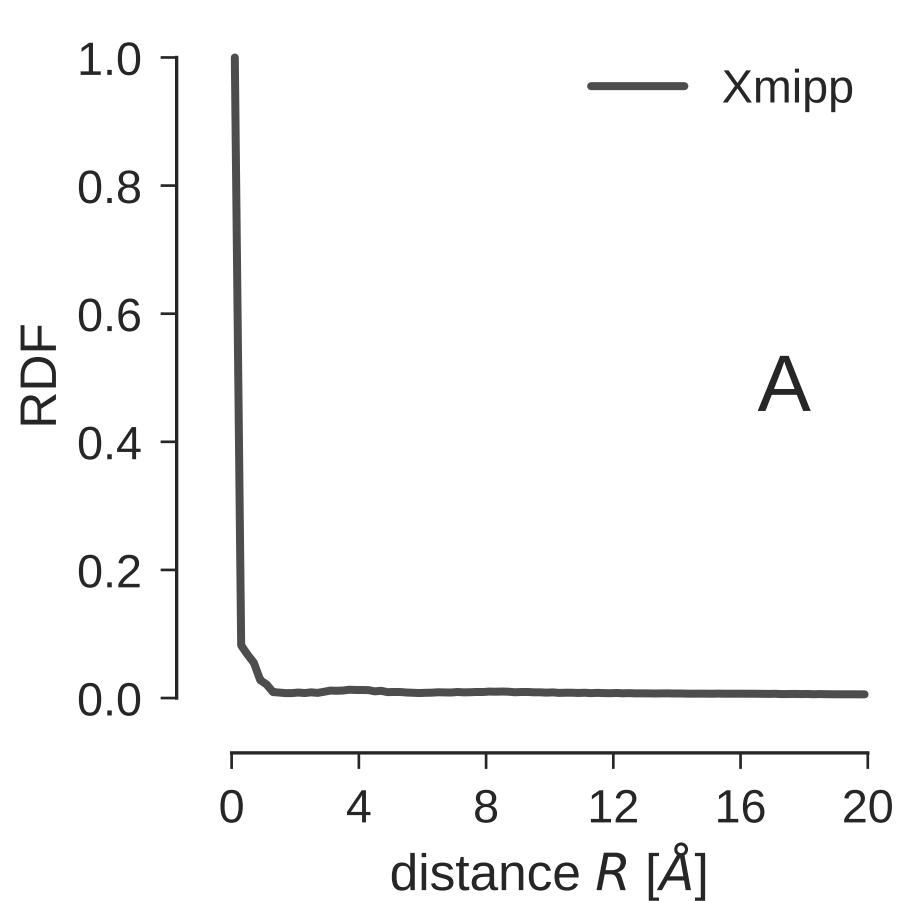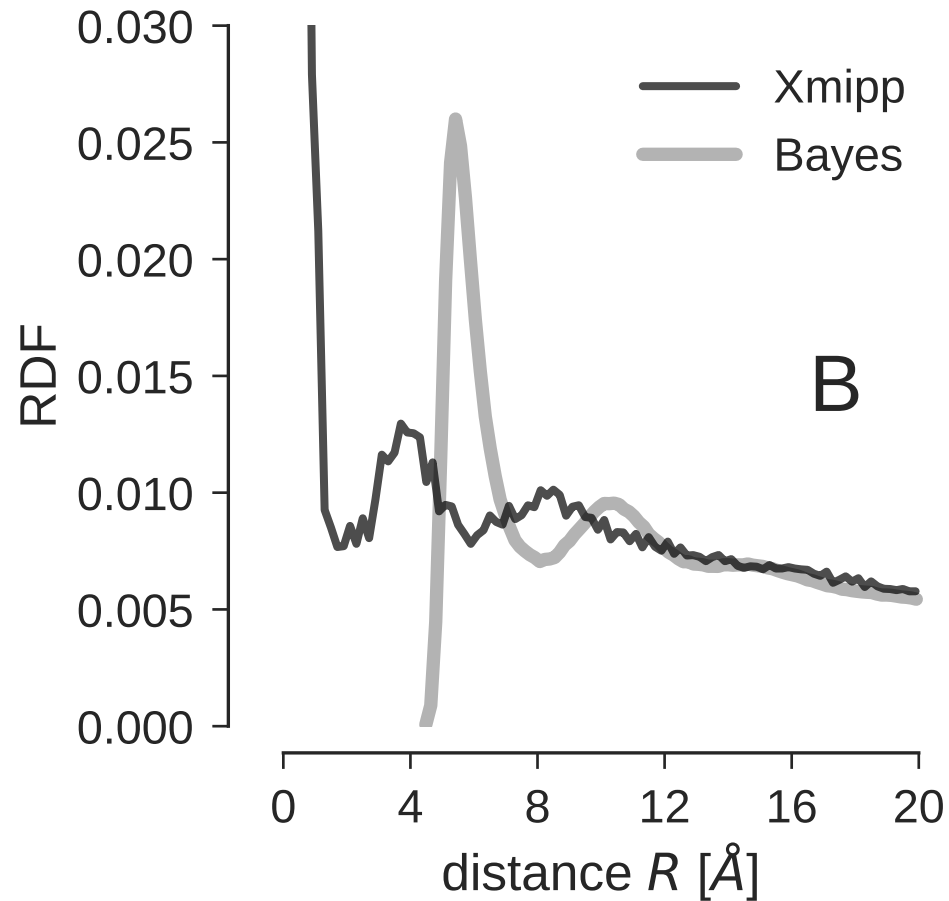

Supplement: S4 Fig — Radial distribution function (RDF) of a CG model obtained from the exosome map using Xmipp’s volume-to-pseudoatom command. (A) The full RDF shows a dominant peak close to zero resulting from a few very small distances. (B) If we zoom into the RDF at larger distances, the RDF shows fluid-like features. However, the first- and second-shell peak are less pronounced than in the RDF resulting from our coarse-graining procedure. (PDF) [file pone.0183057.s004.pdf]
