# Supplementary material for: Data-driven coarse graining of large biomolecular structures
Source: PLoS One. 2017 Aug 17;12(8):e0183057. doi: 10.1371/journal.pone.0183057 (PMC5560709; doi:10.1371/journal.pone.0183057)
Supplement: S1 Appendix — (PDF) [file pone.0183057.s006.pdf]

1 Algorithmic details

1.1 Notation

- $\boldsymbol{x}_n$: Three-dimensional positions of the fine-scale atoms (typically taken from a PDB file). An entire configuration of N fine-scale atoms is stored in the $N \times 3$ matrix $\boldsymbol{x} = \big(\boldsymbol{x}_1, \ldots, \boldsymbol{x}_N\big)'$.
- $\boldsymbol{X}_k$: Positions of CG beads (pseudo-atoms) where $k = 1, \ldots, K$. Typically, we have $N \gg K$. A CG configuration is $\boldsymbol{X} = \big(\boldsymbol{X}_1, \ldots, \boldsymbol{X}_K\big)'$.
- $\boldsymbol{P}_k$: Conjugate momentum variables for the CG positions that will be used in Hamiltonian Monte Carlo.
- Z_{nk}: Binary assignment variable indicating which of the CG particles represents the n-th fine-scale atom.
- $\boldsymbol{Z}$: All assignments collected in a $N \times K$ binary matrix.
- s: Error / resolution of the CG representation.
- $E(\boldsymbol{X}; \boldsymbol{\lambda})$: Force field used to regularize CG configurations.
- $\boldsymbol{\lambda}$: Vector of L force field parameters: $\boldsymbol{\lambda} = \big(\lambda_1, \ldots, \lambda_L\big)'$.
- $\boldsymbol{f}(\boldsymbol{X})$: Vector of features $f_l(\boldsymbol{X})$ such that the force field is a linear combination of the features: $E(\boldsymbol{X}; \boldsymbol{\lambda}) = \sum_l \lambda_l f_l(\boldsymbol{X}) = \langle \boldsymbol{\lambda}, \boldsymbol{f}(\boldsymbol{X}) \rangle$.

1.2 Gaussian mixture model

The Gaussian mixture model can be interpreted as a marginal distribution obtained by summing out the assignment variables:

$$\Pr(\boldsymbol{x}_n \,|\, \boldsymbol{X}, s) = \sum_{Z_{n1} \in \{0,1\}} \cdots \sum_{Z_{nK} \in \{0,1\}} \delta\left(\sum_k Z_{nk} - 1\right) \prod_{k=1}^{K} \left[\frac{1}{K} \mathcal{N}(\boldsymbol{x}_n; \boldsymbol{X}_k, s^2)\right]^{Z_{nk}}. \tag{1}$$

If we use this relation for all atoms, we can write the full likelihood as a marginal distribution over all $N \times K$ assignment variables:

$$\Pr(\boldsymbol{x} \,|\, \boldsymbol{X}, s) = \sum_{\boldsymbol{Z}} \prod_{n=1}^{N} \delta\left(\sum_{k'} Z_{nk'} - 1\right) \prod_{k=1}^{K} \left[\frac{1}{K} \mathcal{N}(\boldsymbol{x}_n; \boldsymbol{X}_k, s^2)\right]^{Z_{nk}} \tag{2}$$

where the sum $\sum_{\boldsymbol{Z}}$ is a short-hand for

$$\sum_{\boldsymbol{Z}} \equiv \sum_{Z_{11} \in \{0,1\}} \cdots \sum_{Z_{1K} \in \{0,1\}} \cdots \sum_{Z_{N1} \in \{0,1\}} \cdots \sum_{Z_{NK} \in \{0,1\}}.$$

The likelihood (2) can be decomposed into a new likelihood which assumes that we know the CG mapping:

$$\Pr(\boldsymbol{x} \,|\, \boldsymbol{X}, \boldsymbol{Z}, s) = \prod_{n=1}^{N} \prod_{k=1}^{K} \left[\mathcal{N}(\boldsymbol{x}_n; \boldsymbol{X}_k, s^2)\right]^{Z_{nk}} \tag{3}$$

and a prior distribution over the binary assignment variables:

$$\Pr(\boldsymbol{Z}) = \prod_n \delta\Big(\sum_k Z_{nk} - 1\Big) \prod_{k=1}^{K} K^{-Z_{nk}} = \prod_n \mathcal{M}(\boldsymbol{Z}_n; 1, \mathbf{1}/K) \tag{4}$$

where $\boldsymbol{Z}_n$ is the n-th row of the assignment matrix (a K-dimensional binary vector) and $\mathbf{1}$ is a K-dimensional vector whose elements are 1. Moreover, we introduced the multinomial distribution in Eq. (4):

$$\mathcal{M}(\boldsymbol{m}; M, \boldsymbol{p}) = \frac{M!}{m_1! \cdots m_K!}\, \delta\big(M - \sum_k m_k\big) \prod_k p_k^{m_k} \tag{5}$$

for counts $m_k \in \{0, \ldots, M\}$ that sum to a total of $M = \sum_k m_k$ and probabilities $p_k \in [0, 1]$, $\sum_k p_k = 1$ represented by a probability vector $\boldsymbol{p}$.

With these choices we have:

$$\Pr(\boldsymbol{x} \,|\, \boldsymbol{X}, s) = \sum_{\boldsymbol{Z}} \Pr(\boldsymbol{x} \,|\, \boldsymbol{X}, \boldsymbol{Z}, s) \times \Pr(\boldsymbol{Z})\,. \tag{6}$$

Instead of summing over all assignment variables analytically, we keep the assignment variables and sample them together with the CG positions using a Gibbs sampler. To see that this is useful, we rewrite the augmented likelihood $\Pr(\boldsymbol{x} \,|\, \boldsymbol{X}, \boldsymbol{Z}, s)$ (Eq. 3):

$$\Pr(\boldsymbol{x} \,|\, \boldsymbol{X}, \boldsymbol{Z}, s) = (2\pi s^2)^{-3N/2} \exp\bigg\{-\frac{1}{2s^2} \sum_k N_k \left[\, \|\boldsymbol{X}_k - \boldsymbol{\mu}_k\|^2 + s_k^2 \,\right]\bigg\} \tag{7}$$

where we introduced the summary statistics

$$N_k = \sum_n Z_{nk}, \quad \boldsymbol{\mu}_k = \frac{1}{N_k} \sum_n Z_{nk} \boldsymbol{x}_n, \quad s_k^2 = \frac{1}{N_k} \sum_n Z_{nk} \|\boldsymbol{x}_n - \boldsymbol{\mu}_k\|^2\,. \tag{8}$$

N_k is the number of atoms that have been assigned to the k-th bead, $\boldsymbol{\mu}_k$ is the center of mass of the assigned fine-scale atoms and s_k the spatial extent of the cluster. The likelihood will pull the beads towards the cluster centers with a harmonic potential whose force constant is N_k/s^2.

1.3 Inference

We use Markov chain Monte Carlo (MCMC) [1] to infer the model parameters $\boldsymbol{X}, \boldsymbol{Z}, s$, and $\boldsymbol{\lambda}$. The general MCMC strategy is a Gibbs sampler [2], which updates groups of

parameters successively by drawing from the conditional posteriors:

$$\boldsymbol{X}|\boldsymbol{Z},s,\boldsymbol{\lambda} \sim \Pr(\boldsymbol{X} \mid \boldsymbol{Z},s,\boldsymbol{\lambda},\boldsymbol{x}) \propto \exp\left\{-\frac{1}{2s^2}\sum_k N_k\|\boldsymbol{X}_k-\boldsymbol{\mu}_k\|^2 - \langle\boldsymbol{\lambda},\boldsymbol{f}(\boldsymbol{X})\rangle\right\} \quad (9)$$

$$\boldsymbol{Z}|\boldsymbol{X},s \sim \Pr\{\boldsymbol{Z}|\boldsymbol{X},s,\boldsymbol{x}\} \propto \prod_n \mathcal{M}(\boldsymbol{Z}_n;1,\boldsymbol{p}_n) \quad (10)$$

$$s^{-2}|\boldsymbol{X},\boldsymbol{Z} \sim \Pr\{s^{-2}|\boldsymbol{X},\boldsymbol{Z},\boldsymbol{x}\} = \mathcal{G}(s^{-2};3N/2,\frac{1}{2}\sum_k N_k[\|\boldsymbol{\mu}_k-\boldsymbol{X}_k\|^2+s_k^2]) \quad (11)$$

$$\boldsymbol{\lambda}|\boldsymbol{X} \sim \Pr\{\lambda|\boldsymbol{X}\} \propto \frac{1}{Z(\boldsymbol{\lambda})}\exp\{-\langle\boldsymbol{\lambda},\boldsymbol{f}(\boldsymbol{X})\rangle\} \quad (12)$$

where we introduced the Gamma distribution

$$\mathcal{G}(t;\alpha,\beta) = \frac{\beta^\alpha}{\Gamma(\alpha)}\,t^{\alpha-1}e^{-\beta t}, \quad t,\alpha,\beta \geq 0 \quad (13)$$

and the assignment probabilities

$$p_{nk} = \frac{\exp\{-\frac{1}{2s^2}\|\boldsymbol{x}_n-\boldsymbol{X}_k\|^2\}}{\sum_{k'}\exp\{-\frac{1}{2s^2}\|\boldsymbol{x}_n-\boldsymbol{X}_{k'}\|^2\}}\,. \quad (14)$$

Steps (10) and (11) are straightforward: We simply have to use standard random number generators of the multinomial distribution and of the Gamma distribution to update the CG mapping Z_{nk} and the precision s^{-2}. Sampling $\boldsymbol{X}$ and $\boldsymbol{\lambda}$ is more challenging.

1.4 Hamiltonian Monte Carlo

The idea of Hamiltonian Monte Carlo (HMC) is to first introduce auxiliary parameters, the *momenta* $\boldsymbol{P}$, and simulate the phase-space distribution:

$$\Pr(\boldsymbol{X},\boldsymbol{P}) \propto \exp\left\{-\frac{1}{2}\|\boldsymbol{P}\|^2 - U(\boldsymbol{X})\right\} = \exp\bigl\{-H(\boldsymbol{X},\boldsymbol{P})\bigr\} \quad (15)$$

where we introduced the Hamiltonian

$$H(\boldsymbol{X},\boldsymbol{P}) = \frac{1}{2}\,\|\boldsymbol{P}\|^2 + U(\boldsymbol{X})\,. \quad (16)$$

Because

$$\int \Pr(\boldsymbol{X},\boldsymbol{P})\,d\boldsymbol{P} \propto \Pr(\boldsymbol{X})\,,$$

we can sample the configurations from $\Pr(\boldsymbol{X})$ by simulating the phase space ensemble $\exp\{-H(\boldsymbol{X},\boldsymbol{P})\}$. This is achieved by combining MD simulation with a Metropolis step.

The idea is to start from a current configuration $\boldsymbol{X}(0)$ and generate initial momenta by drawing from a K three-dimensional standard Normal distributions:

$$\boldsymbol{P}_k(0) \sim \mathcal{N}(\boldsymbol{P}_k(0);\boldsymbol{0},1)\,. \quad (17)$$

The initial positions $\boldsymbol{X}_k(0)$ could be the cluster centers $\boldsymbol{\mu}_k$ defined in Eq. (8) or the previous configuration obtained during Gibbs sampling. We generate a proposal state in phase space $(\boldsymbol{X}(\tau), \boldsymbol{P}(\tau))$ by integrating Hamilton's equations of motion

$$\dot{\boldsymbol{X}} = \boldsymbol{P}, \quad \dot{\boldsymbol{P}} = -\boldsymbol{\nabla} U(\boldsymbol{X}) \tag{18}$$

until an integration time τ. We accept $(\boldsymbol{X}(\tau), \boldsymbol{P}(\tau))$ as a new state with probability

$$\Pr(\boldsymbol{X}(\tau), \boldsymbol{P}(\tau) \,|\, \boldsymbol{X}(0), \boldsymbol{P}(0)) = \min\Big\{1, \exp\big\{H(\boldsymbol{X}(0), \boldsymbol{P}(0)) - H(\boldsymbol{X}(\tau), \boldsymbol{P}(\tau))\big\}\Big\}.$$

If we could integrate Hamilton's equations of motion (18) exactly, we would always accept because the Hamiltonian is conserved under Hamiltonian dynamics. However, for realistic systems we have to use numerical methods to integrate Hamilton's equations of motion.

A popular integrator for Hamilton's equations of motion is the *leapfrog* algorithm. It uses a stepsize ϵ and consists of alternating momentum and position update:

$$\begin{aligned}
\boldsymbol{P}(t+\epsilon/2) &= \boldsymbol{P}(t) - \epsilon/2 \boldsymbol{\nabla} U(\boldsymbol{X}(t)) \\
\boldsymbol{X}(t+\epsilon) &= \boldsymbol{X}(t) + \epsilon \boldsymbol{P}(t+\epsilon/2) \\
\boldsymbol{P}(t+\epsilon) &= \boldsymbol{P}(t+\epsilon/2) - \epsilon/2 \boldsymbol{\nabla} U(\boldsymbol{X}(t+\epsilon))
\end{aligned}$$

1.5 Modeling cryo-EM maps with bead models

It is straight forward to extend our CG graining algorithm to density maps obtained with cryo-EM. Let us assume that the structure for which we would like to find a CG representation is given in the form of a density map with real-valued intensities ρ_n at grid points $\boldsymbol{x}_n$. Our model for the density at position $\boldsymbol{x}$ is again a mixture of Gaussians centered at the unknown CG positions:

$$\rho(\boldsymbol{x}; \boldsymbol{X}, s) = \sum_k \frac{1}{(2\pi s^2)^{3/2}} \exp\Big\{-\frac{1}{2s^2}\|\boldsymbol{x} - \boldsymbol{X}_k\|^2\Big\}$$

We use the Poisson distribution as the likelihood of observing the experimental map:

$$\Pr(\rho \,|\, \boldsymbol{X}, s, \alpha) = \prod_n [\alpha \rho(\boldsymbol{x}_n; \boldsymbol{X}, s)]^{\rho_n} e^{-\alpha\rho(\boldsymbol{x}_n; \boldsymbol{X}, s)} / (\rho_n!)$$

where $\alpha > 0$ is an unknown scaling parameter. The resulting log likelihood is:

$$\begin{aligned}
\log \Pr(\rho \,|\, \boldsymbol{X}, s, \alpha) &= \sum_n \rho_n \log[\alpha\rho(\boldsymbol{x}_n; \boldsymbol{X}, s)] - \alpha\rho(\boldsymbol{x}_n; \boldsymbol{X}, s) \\
&\approx \sum_n \rho_n \log \rho(\boldsymbol{x}_n; \boldsymbol{X}, s) + \log\alpha \sum_n \rho_n - K\alpha
\end{aligned} \tag{19}$$

where we assumed $\sum_n \rho(\boldsymbol{x}_n; \boldsymbol{X}, s) \approx \int \rho(\boldsymbol{x}; \boldsymbol{X}, s)\, d\boldsymbol{x} = K$. Let us simplify the $\boldsymbol{X}$ dependent part of the log likelihood:

$$\sum_n \rho_n \log \rho(\boldsymbol{x}_n; \boldsymbol{X}, s) = \sum_n \rho_n \log \sum_k \frac{1}{(2\pi s^2)^{3/2}} \exp\left\{-\frac{1}{2}\|\boldsymbol{x}_n - \boldsymbol{X}_k\|^2/s^2\right\}$$
$$\geq -\frac{1}{2}\sum_n \sum_k \rho_n Z_{nk} \left[\|\boldsymbol{x}_n - \boldsymbol{X}_k\|^2/s^2 + 3\log(2\pi s^2)\right]$$

where we introduced the assignment variables:

$$Z_{nk} = \frac{\exp\{-\frac{1}{2s^2}\|\boldsymbol{x}_n - \boldsymbol{X}_k\|^2\}}{\sum_{k'} \exp\{-\frac{1}{2s^2}\|\boldsymbol{x}_n - \boldsymbol{X}_{k'}\|^2\}}$$

and used Jensen's inequality to derive a lower bound of the log likelihood. Maximization of the lower bound results in an estimate of the CG positions:

$$\boldsymbol{\mu}_k = \frac{1}{\sum_n \rho_n Z_{nk}} \sum_n \rho_n Z_{nk}\, \boldsymbol{x}_n \tag{20}$$

and of the precision of the representation:

$$s^2 = \frac{1}{3\sum_n \rho_n} \sum_{n,k} \rho_n Z_{nk}\, \|\boldsymbol{x}_n - \boldsymbol{X}_k\|^2\,. \tag{21}$$

The CG energy has the same functional form as before (Eq. **??**):

$$\frac{1}{2s^2}\sum_k N_k \|\boldsymbol{\mu}_k - \boldsymbol{X}_k\|^2, \quad N_k = \sum_n \rho_n Z_{nk}$$

where the only difference is that the fine-grained positions $\boldsymbol{x}_n$ are weighted by their density values ρ_n.

References

[1] J. S. Liu. *Monte Carlo strategies in scientific computing.* Springer, 2001.

[2] S. Geman and D. Geman. Stochastic Relaxation, Gibbs Distributions, and the Bayesian Restoration of Images. *IEEE Trans. PAMI*, 6(6):721–741, 1984.
