# Supplementary material for: Data-driven coarse graining of large biomolecular structures
Source: PLoS One. 2017 Aug 17;12(8):e0183057. doi: 10.1371/journal.pone.0183057 (PMC5560709; doi:10.1371/journal.pone.0183057)
Supplement: S5 Fig — See the caption of Fig 6 for more details. The insets show that CG models that served as an input for the anisotropic network analysis. The lower right panel shows the overlap distribution of the Cα model for comparison. (PDF) [file pone.0183057.s005.pdf]

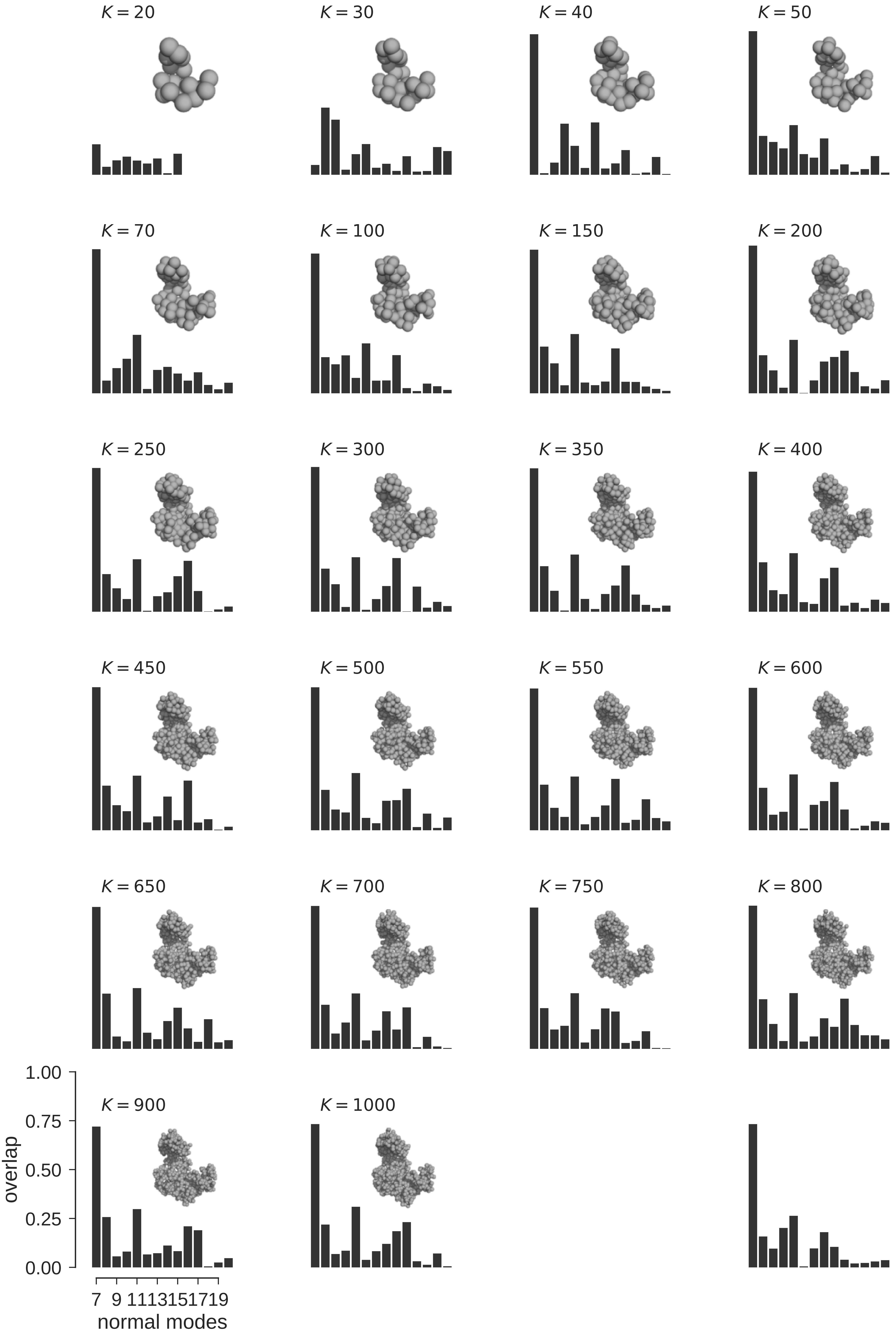

$K = 20$
$K = 30$
$K = 40$
$K = 50$
$K = 70$
$K = 100$
$K = 150$
$K = 200$
$K = 250$
$K = 300$
$K = 350$
$K = 400$
$K = 450$
$K = 500$
$K = 550$
$K = 600$
$K = 650$
$K = 700$
$K = 750$
$K = 800$
$K = 900$
$K = 1000$
1.00
0.75
0.50
0.25
0.00
overlap
7
9
111315171719
normal modes
